# *In Vivo* Conditions to Identify Prkci Phosphorylation Targets Using the Analog-Sensitive Kinase Method in Zebrafish

**DOI:** 10.1371/journal.pone.0040000

**Published:** 2012-06-29

**Authors:** Elena Cibrián Uhalte, Marieluise Kirchner, Nicole Hellwig, Jasmina J. Allen, Stefan Donat, Kevan M. Shokat, Matthias Selbach, Salim Abdelilah-Seyfried

**Affiliations:** 1 Max Delbrück Center for Molecular Medicine, Berlin, Germany; 2 Howard Hughes Medical Institute, University of California San Francisco, San Francisco, California, United States of America; 3 Department of Cellular and Molecular Pharmacology, University of California San Francisco, San Francisco, California, United States of America; National University of Singapore, Singapore

## Abstract

Protein kinase C iota is required for various cell biological processes including epithelial tissue polarity and organ morphogenesis. To gain mechanistic insight into different roles of this kinase, it is essential to identify specific substrate proteins in their cellular context. The analog-sensitive kinase method provides a powerful tool for the identification of kinase substrates under *in vivo* conditions. However, it has remained a major challenge to establish screens based on this method in multicellular model organisms. Here, we report the methodology for *in vivo* conditions using the analog-sensitive kinase method in a genetically-tractable vertebrate model organism, the zebrafish. With this approach, kinase substrates can uniquely be labeled in the developing zebrafish embryo using bulky ATPγS analogs which results in the thiophosphorylation of substrates. The labeling of kinase substrates with a thiophosphoester epitope differs from phosphoesters that are generated by all other kinases and allows for an enrichment of thiophosphopeptides by immunoaffinity purification. This study provides the foundation for using the analog-sensitive kinase method in the context of complex vertebrate development, physiology, or disease.

## Introduction

Phosphorylation is a protein modification that is essential for almost all aspects of cell biology. Kinases that catalyze this post-translational modification are an abundant group of enzymes with promiscuous substrate specificity and a common requirement for ATP. For this reason, the identification of specific substrate proteins of particular kinases has remained a tedious challenge and traditionally involves *in vitro* phosphorylation assays with candidate substrates. However, *in vitro* assays often generate false-positive results and are inferior to *in vivo* screening conditions in which the kinase of interest localizes within the correct subcellular compartment and is associated with endogenous binding partners that modulate its activity and affinity towards substrate proteins. The analog-sensitive kinase method utilizes *in vivo* conditions for substrate identification but has never been employed in a multicellular model organism [1].

The atypical protein kinase C (aPKC) family consists of serine/threonine kinases with essential cellular functions in cell polarity and organ morphogenesis, cell migration, apoptosis and proliferation (reviewed in [2]–[5]). Increasing evidence also points at an involvement of aPKCs in the promotion of carcinogenesis *in vitro* and *in vivo* [reviewed in [6]]. Among other proteins, aPKCs are core components of the apical Partition defective 6 (Pard6)-aPKC protein complex which is composed of several PDZ domain containing proteins and is required for the establishment of epithelial apicobasal polarity in many systems (reviewed in [4], [7]). The unique N-terminal regulatory domain of aPKCs which contains a Phox Bem1 (PB1) domain mediates direct interactions with the polarity protein Pard6 which in turn modulates aPKC activity, or with the small GTPases Rac1 and Cdc42 [8]. The zebrafish *heart and soul* locus encodes Prkci, one of two aPKCs expressed in this organism [9], [10]. Consistent with a function in apicobasal cell polarity, zebrafish mutants lacking Prkci show defective formation and maintenance of several embryonic epithelia and abnormal heart morphogenesis [9], [11], [12]. Prkci function in cellular polarity and organ morphogenesis requires its catalytic activity [12].

To date, only a small number of substrate proteins of Prkci in the context of cellular polarity, proliferation control or apoptosis have been identified in any organism, and the molecular mechanisms of their interaction with aPKCs have been convincingly demonstrated for only a few of them [13]–[19]. In addition to its auto-phosphorylation [3], potentially relevant substrate proteins of aPKC activity in the context of cellular polarity are Par3 [17], [20], [21], Numb [22], [23], Miranda [24], Frizzled 1 [25], Partner of inscuteable [26], and GSK-3β [8]. To further elucidate the mechanistic relevance of aPKCs during development and in various cell biological processes it is necessary to identify phosphorylation targets in an unbiased manner and under *in vivo* conditions.

We have established conditions for a chemical genetics screen using the analog-sensitive kinase method to identify phosphorylation targets of Prkci during zebrafish development. This methodology opens the way for the identification and functional characterization of specific substrates in their normal subcellular context which is essential for a mechanistic understanding of how Prkci affects divergent cellular processes.

## Results

### Design of an Analog-sensitive Prkci

**Figure 1:**
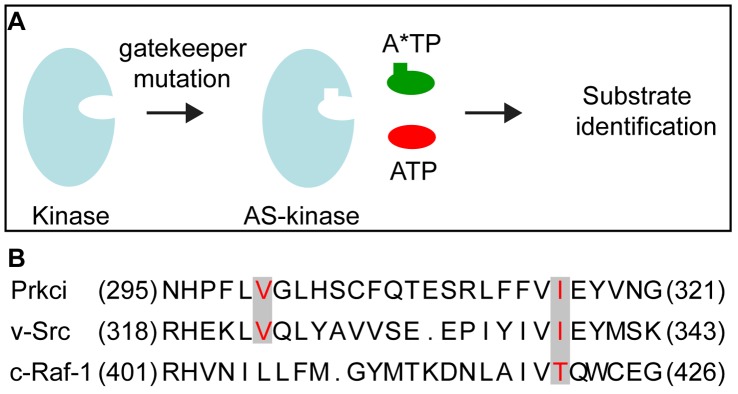
Design of the analog-sensitive Prkci. (**A**) A space-creating mutation (“gatekeeper mutation”) is introduced into the kinase ATP binding pocket which allows the analog-sensitive (AS) kinase mutant to accept a bulky ATP analog (A*TP) required for the chemical genetic identification of kinase substrates [modified after [36]]. (**B**) Alignment of the primary sequence of the ATP binding pocket within the kinase domains of Prkci, v-Src and c-Raf. The residues in red correspond to the amino acids mutated in v-Src [1], c-Raf-1 [28], and Prkci to enlarge the ATP binding pocket.

We established a screening approach with the aim to identify Prkci phosphorylation targets in a multicellular model organism. The straightforward screening method using analog-sensitive kinases utilizes an environment that is similar to the *in vivo* state of the kinase. In principle, the method is based on engineering a mutant kinase that accepts bulky ATP or ATPγS analogs such as *N* ^6^-benzyl ATP or *N* ^6^-benzyl ATPγS. The ATP analog-sensitive kinase is generated by replacing a large hydrophobic group within the ATP binding pocket (the “gatekeeper” residue) with a smaller residue thereby enlarging the binding pocket which allows the kinase to accept bulkier ATP analogs (Fig. 1A). The specificity of this approach has been demonstrated for various kinases, e.g. by utilizing a modified v-Src, c-Raf-1, or AMPKα2 for the identification of novel substrates [1], [27], [28].

To identify an ATP binding site “gatekeeper” residue within the Prkci ATP binding pocket, we selected suitable residues for site-directed mutagenesis based on the high evolutionary conservation of the ATP binding site in different protein kinases, including v-Src [1] and c-Raf-1 [28] that had successfully been engineered to utilize *N* ^6^-benzyl ATP. The comparison of the primary sequence of Prkci, c-Src, and c-Raf-1 kinase domains suggested Val_300_ and Ile_316_ of Prkci to be the most likely “gatekeeper” residues and therefore to be the most suitable targets for site-directed mutagenesis (Fig. 1B).

### Mutant Prkci^I316A^ has Normal *in Vivo* Biological Activity

**Figure 2:**
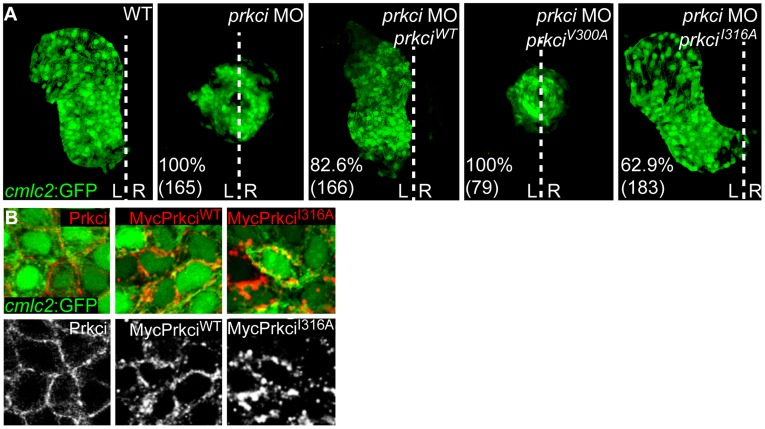
Mutant Prkci^I316A^ has normal *in vivo* biological activity. (**A**) Reconstruction of confocal Z-stack sections of embryonic hearts at 28–30 hpf. Transgenic Tg[*cmlc2:GFP*]^twu34^ one-cell stage embryos were injected with *prkci* MO alone or together with mRNA encoding Prkci^WT^ or analog-sensitive mutant forms of Prkci. Whereas the wild-type heart elongates into a heart tube and towards the left during cardiac jogging, heart development arrests at the cone stage and the heart remains at the embryonic midline in *prkci* morphants. In functional rescue experiments, injection of *prkci*MO together with mRNA encoding HisMyc-Prkci^WT^ or Prkci^I316A^ rescues heart tube elongation. In comparison, the analog-sensitive mutant form Prkci^V300A^ fails to rescue heart tube formation in *prkci* morphants. Percentiles indicate the occurrence of the most common phenotype as depicted in the images and numbers show the total of embryos tested. White dotted line indicates the embryonic midline. L, left; R, right. (**B**) Membrane localization of endogenous Prkci detected with an anti-Prkci antibody and exogenous Prkci^WT^ or Prkci^I316A^ in zebrafish cardiomyocytes detected with an anti-Myc antibody. Images are confocal reconstructions of single Z-stack sections of embryonic hearts marked by the transgenic reporter Tg[*cmlc2:GFP*]*^twu34^* at 28–30 hpf. Expression of exogenous HisMyc-Prkci^WT^ or HisMyc-Prkci^I316A^ in cardiomyocytes reveals that both recombinant proteins localize to the cell membrane.

One stringent requirement for an analog-sensitive kinase is the conservation of its biological properties which includes normal *in vivo* functionality and substrate specificity. We therefore tested whether the Prkci^V300A^ or Prkci^I316A^ mutants with altered ATP binding pockets had normal biological activity. Loss of Prkci has well-characterized epithelial and organ morphogenesis defects that involve cardiac malformations and a defective neuroepithelium [9], [11], [12]. In functional rescue experiments, we co-injected at the one-cell stage mRNA encoding either Prkci^WT^ or one of the two mutant Prkci proteins together with an antisense oligonucleotide morpholino (MO) for knockdown of endogenous Prkci. Whereas expression of Prkci^I316A^ allowed normal cardiac development (63% of *prkci* morphant embryos rescued, n = 183) which was almost as efficient as Prkci^WT^ expression (83% of prkci morphants rescued, n = 166), the Prkci^V300A^ mutant was not biologically active based on the appearance of *prkci* morphant cardiac phenotypes among all injected embryos (Fig. 2A) [9].

Because the Prkci^I316A^ mutant was biologically fully functional within the heart, we anticipated that this mutant kinase should correctly localize within myocardial cells. Indeed, expression by injection at the one-cell stage of mRNA encoding Myc-tagged Prkci^I316A^ resulted in the correct localization of the mutant protein to the cell membrane, similar to Myc-tagged Prkci^WT^ or endogenous Prkci (Fig. 2B). That overexpression of Prkci^WT^ or Prkci^I316A^ did not cause any ectopic phenotypes strongly suggests substrate specificity (see below). Together, our results indicated that Prkci^I316A^ is biologically functional and predominantly localizes to the correct subcellular compartment at the cell membrane, which makes this mutant kinase a strong candidate for a chemical genetic screen.

### Mutant Prkci^I316A^ can use Bulky ATP Analogs

**Figure 3:**
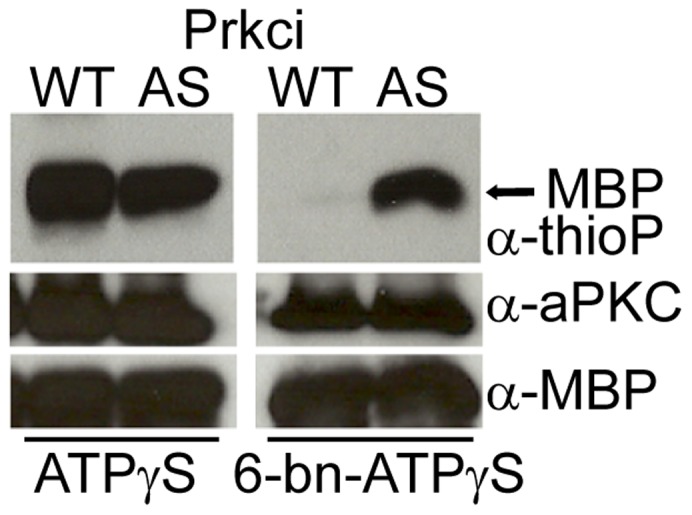
Mutant Prkci^I316A^ uses bulky *N^6^*-benzyl ATPγS. Kinase reaction with Myelin Basic Protein (MBP) and ATPγS or *N^6^*-benzyl ATPγS (6-bn- ATPγS), followed by PNBM alkylation. In comparison, only mutant Prkci^I316A^ efficiently utilizes *N^6^*-benzyl ATPγS to thiophosphorylate MBP. Labeled MBP is detected by Western blot analysis with rabbit monoclonal anti-thiophosphoester antibody (α-thioP).

We next tested whether Prkci^I316A^ could utilize bulky ATP analogs by assaying the phosphorylation efficiency and specificity of several alkylated ATPγS analogs in *in vitro* kinase assays. ATPγS was utilized instead of regular ATP to ensure a transfer of a phosphorothioate moiety to the phosphoacceptor hydroxyl groups of respective substrates. The substitution of sulfur in place of oxygen generates unique thiophosphorylated epitopes that, when alkylated with *p*-nitrobenzyl mesylate (PNBM), generate thiophosphoester epitopes which can be recognized by a specific monoclonal antibody [29], [30]. Using the baculovirus system in Sf9 insect cells, we produced the recombinant kinases and first confirmed that Prkci^WT^ and Prkci^I316A^ could utilize ATPγS to phosphorylate Myelin Basic Protein (MBP) as substrate and that this modification could efficiently be detected with the anti-thiophosphoester antibody (Fig. 3). However, whereas Prkci^WT^ accepted ATPγS, it could not utilize any of the tested *N^6^*-alkylated ATPγS analogs (*N^6^*-benzyl-, *N^6^*-phenethyl-, or *N^6^*-cyclopentyl- ATPγS) [30] as assessed on Western blot upon the *in vitro* kinase assay. In contrast, Prkci^I316A^ most efficiently utilized *N* ^6^-benzyl ATPγS (Fig. 3) whereas the other two bulky ATPγS analogs were apparently not efficiently utilized (data not shown). Taken together, Prkci^I316A^ had a normal biological function in the *in vivo* context and exerted catalytic activity using a bulky *N^6^*-benzyl-ATP analog. Hence, Prkci^I316A^ fulfilled the basic requirements required to identify Prkci phosphorylation targets.

### Evidence for Prkci^I316A^-mediated Thiophosphorylation in the Zebrafish Embryo

Two principal methods have been used for the enrichment of thiophosphorylated proteins in *in vitro* phosphorylation assays and in cell culture systems but not yet in any model organism. The “covalent capture” approach is based on the enrichment of thiophosphate-tagged substrates with iodoacetyl-agarose and subsequent analysis by mass spectrometry [31]. An alternative approach is based on “immunoaffinity purification” enrichment using the anti-thiophosphoester antibody, followed by mass spectrometry of enriched peptides [27], [30]. Both methods require the utilization of ATPγS analogs for the unique labelling of substrate proteins.

**Figure 4:**
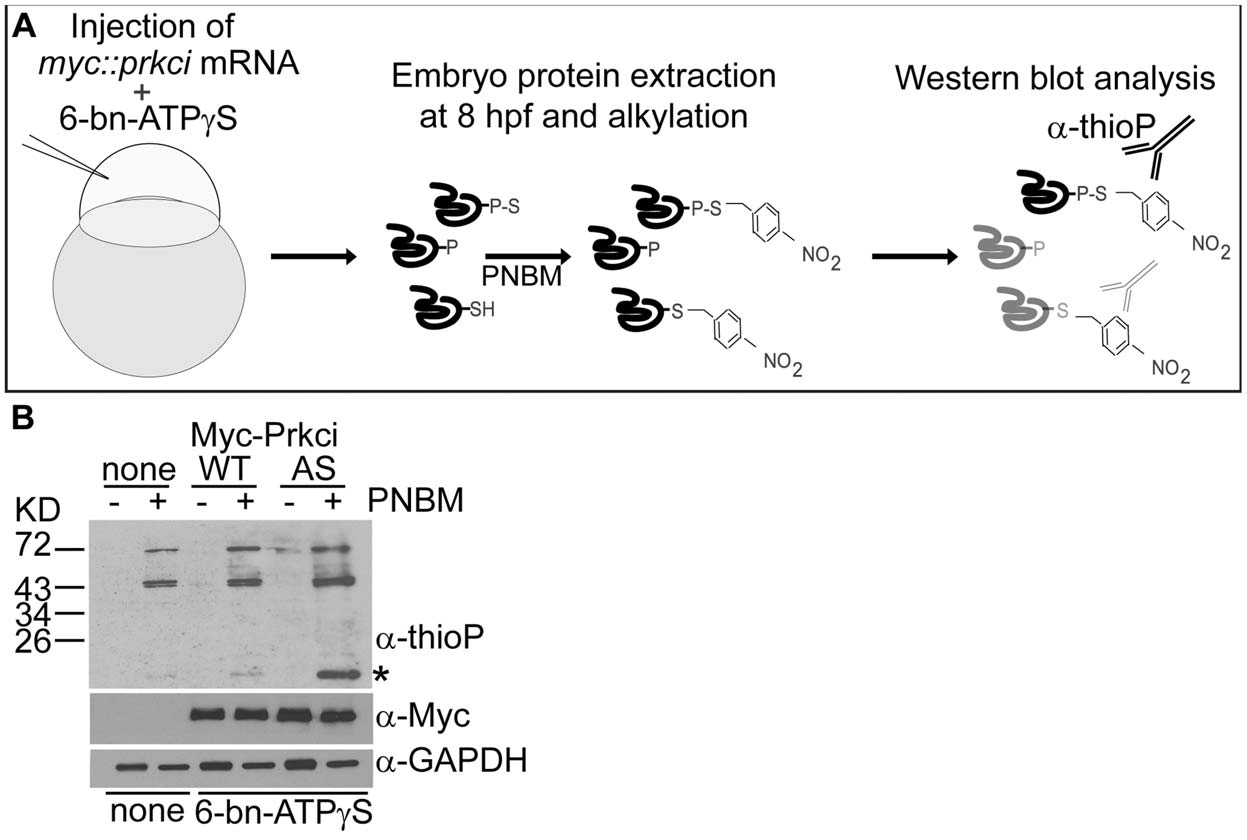
Thiophosphorylation of substrate proteins by Prkci^I316^ in the zebrafish embryo. (**A**) Schematic diagram of the *in vivo* labeling method for the selective labeling of Prkci^I316A^ substrates during zebrafish development. (**B**) *In vivo* thiophosphorylation in zebrafish embryos injected at the one-cell stage with 200 μM *N^6^*-benzyl-ATPγS (6-bn-ATPγS) and mRNA encoding either Prkci^WT^ or Prkci^I316A^ (AS). Western blot analysis with rabbit monoclonal anti-thiophosphoester (α-thioP) C51-8 antibody (Epitomics) of 80% epiboly (6–8hpf) samples alkylated with 2.5 mM PNBM reveals a selectively labeled protein in the Prkci^I316A^ (AS) sample (asterisk).

One preeminent challenge of using ATPγS analogs in *in vivo* approaches is the potential toxicity of thiophosphates since such protein modifications cannot be removed by phosphatases. We first tested the toxicity of *N* ^6^-benzyl ATPγS analog by injecting different concentrations (∼1 nL injection volume) into wild-type embryos and found that a concentration of 200 µM of *N^6^*-benzyl ATPγS was the maximal concentration that could be injected into one-cell stage embryos without affecting development (200 µM: 93.3% of embryos developed normally, n = 45; 250 µM: 47.8% of embryos developed normally, n = 23; 500 µM: 37.2% of embryos developed normally, n = 43). We next tested the feasibility of applying this approach in the zebrafish embryo by optimizing the conditions for introducing *N* ^6^-benzyl ATPγS together with mRNA encoding Prkci^I316A^ by microinjection at the one-cell stage (Fig. 4A). With the concentration of 200 µM *N* ^6^-benzyl ATPγS (^∼^1 nL injection volume), we observed that most embryos survived up to 32 hours post fertilization (hpf) (97.2% of Prkci^I316A^ expressing embryos developed normally, n = 320), which was comparable with embryos co-injected with *N* ^6^-benzyl ATPγS and mRNA encoding Prkci^WT^ (96.5% of embryos developed normally, n = 261), or with non-injected control embryos (96.7% of embryos developed normally, n = 390). Therefore, *N* ^6^-benzyl ATPγS in the range of physiological ATP levels is compatible with normal zebrafish embryogenesis.

To assess whether thiophosphorylation had occurred in the developing zebrafish embryo, such *in vivo* thiophosphorylated 8 hpf extracts were alkylated using PNBM, resolved by SDS-PAGE, and Western blots probed with the anti-thiophosphoester antibody. This analysis revealed that thiophosphorylation of substrates had indeed occurred in the zebrafish embryo and that Prkci^I316A^ had catalyzed the selective labelling of at least one putative substrate (Fig. 4B).

## Discussion

This study outlines the methodology required for an *in vivo* screening approach using an analog-sensitive kinase in a multi-cellular model organism. Our work demonstrates the conditions for *in vivo* thiophosphoester labeling of substrates using an analog-sensitive kinase and bulky *N* ^6^-benzyl ATPγS analogs. Injection of physiological levels of bulky *N* ^6^-benzyl ATPγS does not interfere with zebrafish development even though thiophosphorylations are largely irreversible. Viability of zebrafish embryos under such conditions indicates that only a fraction of substrate proteins is modified by the analog-sensitive kinase and such *in vivo* thiophosphorylations are detectable on Western blots. That thiophosphorylations are also detected in Prkci^WT^ samples is due to unspecific utilization of *N* ^6^-benzyl ATPγS by other enzymes [31] and to the partial degradation of *N* ^6^-benzyl ATPγS to ATPγS which can be utilized by other kinases. These contaminations with unspecific thiophosphoylations highlight the need to immunopurify embryonic extracts and to perform comparative mass spectrometric analyses for substrate identification.

The method for the enrichment and identification of novel putative kinase targets by immunoaffinity purification with an anti-thiophosphoester antibody is well-established [27], [30]. Combining this methodology with the working protocol outlined in our study will soon provide unprecedented insight into kinase signaling in multi-tissue encompassing developmental processes, the regulation of different physiological conditions, or in disease processes involving aberrant kinase signaling. Taken together, our work provides the ground for similar approaches using the analog-sensitive kinase method in this and other multicellular model organisms.

## Materials and Methods

### Fish Maintenance and Stocks

Zebrafish were maintained at standard conditions [32]. Embryos were kept in egg water (60 µg/ml Instant Ocean Sea Salts, Aquarium Systems Inc., USA) and staged at 28.5°C [33]. The following fish strains were used: AB (wild-type), Tg[*cmlc2:GFP*]*^twu34^* [34].

### RNA and Antisense Oligonucleotide Morpholino Injections

Constructs were transcribed using the SP6 mMessage mMachine kit (Ambion). Tg[*cmlc2:GFP*]*^twu34^* embryos were injected with 2.5 ng of *prkci* MO [12]. For rescue experiments 100 pg of mRNAs were injected. The heart morphology was assessed at 24hpf. Data presented are the means of at least 2 independent experiments. For *in vivo* labeling, 200 pg of mRNA encoding HisMyc-Prkci^WT^ or HisMyc-Prkci^I316A^ mRNA were injected at the one-cell stage.

The antisense oligonucleotide morpholino was purchased from Gene Tools, LLC, USA. *prkci* MO (5′→ 3′): TGTCCCGCAGCGTGGGCATTATGGA [12].

### DNA Constructs and Site-directed Mutagenesis

Both constructs encoding wt and mutant forms of Prkci were produced by PCR amplification from a full length cDNA template, pCS2+ *HisMyc::prkci* [12]. Site directed mutagenesis was performed using the QuickChange™ XL Site-Directed mutagenesis kit (Stratagene). Primer sequences are available upon request.

### Protein Extraction from Zebrafish Embryos

Zebrafish embryos protein extraction was performed as previously described [35]. Embryos were dechorionated with pronase solution in E2 medium in Petri dishes coated with 1% agarose. After washes with E2 medium, embryos were transferred to 1.5 ml tubes. The yolk was disrupted by pipetting with a 1 ml pipet and vortexing for 30 seconds at 1100 rpm in deyolking buffer. Embryos were pelleted several times after washes in washing buffer at 3000 rpm for 30 seconds. Pelleted embryos were homogenized in the appropriated lysis buffer depending on the following experiment.

### Recombinant Protein Expression in Insect Cells

To express recombinant Prcki^WT^ or Prkci^I316A^ in Sf9 insect cells (Sigma), Bac-to-Bac® Baculovirus Expression System (Invitrogen) was used according to the manufacturers’ protocol. *HisMyc::prkci^WT^* and *HisMyc::prkci^I316^* cDNAs were cloned into pFastBac^TM^1. Primer sequences are available upon request.

### Whole-mount Immunohistochemistry and Confocal Imaging

Whole-mount antibody stainings were performed as previously described [9]. The following antibodies were used: rabbit anti-aPKC (1∶100, Santa Cruz SC-216), mouse anti-Myc (1∶200, Invitrogen), goat anti-rabbit RRX (1∶250, Jackson ImmunoResearch Laboratories), goat anti-mouse Cy5 (1∶250, Jackson ImmunoResearch Laboratories). For imaging, samples were embedded in SlowFade® Gold antifade reagent (Invitrogen) under a binocular microscope (Leica). Confocal images were obtained with a Zeiss LSM 510 Meta confocal microscope using 40X or 63X objectives. Zeiss LSM 510 software was used to record images. Images were processed using Photoshop (Adobe).

### *In vitro* kinase Assay

For *in vitro* kinase assays 50 µg of MBP were incubated at 30°C for 30 minutes with 5µg of recombinant HisMyc-Prcki^WT^ or HisMyc-Prkci^I316A^ and 500 µM ATPγS or *N* ^6^-benzyl ATPγS (BioLog, B072-05) in kinase buffer [25mM Tris-HCl, pH 7.5, 25 mM NaCl, 10 mM MgCl_2_, 1 mM EGTA, protease inhibitor cocktail (Roche)]. The other bulky ATP analogs (*N^6^*-phenethyl-ATPγS, *N^6^*-cyclopentyl-ATPγS) were generated as described [30]. The kinase reaction was stopped by adding 4x SDS loading buffer and boiling at 95°C for 5 minutes. Samples were analyzed by Western blot using the following antibodies: mouse anti-Myc (1∶1000, Invitrogen), anti-thiophosphoester rabbit polyclonal antibody (1∶5000 Epitomics), rabbit anti-GAPDH (1∶1000), goat anti-mouse HRP (1∶5000, Jackson ImmunoResearch), goat anti-rabbit HRP (1∶10000, Jackson ImmunoResearch).

### *In Vivo* Thiophosphorylation in Zebrafish Embryos

For *in vivo* thiophosphorylation 200 pg of mRNA encoding HisMyc-Prkci^WT^ or HisMyc-Prkci^I316A^ were injected into the yolk at the one-cell stage together with approximately 1 nL of 200 µM *N* ^6^-benzyl ATPγS. Embryonic protein extracts were prepared at 8 hpf, and pelleted embryos were homogenized in RIPA buffer. Alkylation was perfomed for 2 hours at RT with 2.5 mM PNBM [30]. Samples were analyzed by Western blot using anti-thiophosphoester rabbit monoclonal C51-8 antibody (1∶5000 Epitomics).

## References

[1] Liu Y, Shah K, Yang F, Witucki L, Shokat KM (1998). Engineering Src family protein kinases with unnatural nucleotide specificity.. Chem Biol.

[2] Bakkers J, Verhoeven MC, Abdelilah-Seyfried S (2009). Shaping the zebrafish heart: from left-right axis specification to epithelial tissue morphogenesis.. Dev Biol.

[3] Hirai T, Chida K (2003). Protein kinase Cζ (PKCζ): activation mechanisms and cellular functions.. J Biochem.

[4] St. Johnston D, Sanson B (2011). Epithelial polarity and morphogenesis.. Curr Opin Cell Biol.

[5] Suzuki A, Akimoto K, Ohno S (2003). Protein kinase C lambda/iota (PKClambda/iota): a PKC isotype essential for the development of multicellular organisms.. J Biochem.

[6] Murray NR, Kalari KR, Fields AP (2011). Protein kinase Ciota expression and oncogenic signaling mechanisms in cancer.. J Cell Physiol.

[7] Suzuki A, Ohno S (2006). The PAR-aPKC system: lessons in polarity.. J Cell Sci.

[8] Etienne-Manneville S, Hall A (2003). Cell polarity: Par6, aPKC and cytoskeletal crosstalk.. Curr Opin Cell Biol.

[9] Horne-Badovinac S, Lin D, Waldron S, Schwarz M, Mbamalu G (2001). Positional cloning of heart and soul reveals multiple roles for PKC lambda in zebrafish organogenesis.. Curr Biol.

[10] Peterson RT, Mably JD, Chen JN, Fishman MC (2001). Convergence of distinct pathways to heart patterning revealed by the small molecule concentramide and the mutation *heart-and-soul*.. Curr Biol.

[11] Horne-Badovinac S, Rebagliati M, Stainier DY (2003). A cellular framework for gut-looping morphogenesis in zebrafish.. Science.

[12] Rohr S, Bit-Avragim N, Abdelilah-Seyfried S (2006). Heart and soul/Prkci and Nagie oko/Mpp5 regulate myocardial coherence and remodeling during cardiac morphogenesis.. Development.

[13] Betschinger J, Mechtler K, Knoblich JA (2003). The Par complex directs asymmetric cell division by phosphorylating the cytoskeletal protein Lgl.. Nature.

[14] Betschinger J, Eisenhaber F, Knoblich JA (2005). Phosphorylation-induced autoinhibition regulates the cytoskeletal protein Lethal (2) giant larvae.. Curr Biol.

[15] Galli M, Munoz J, Portegijs V, Boxem M, Grill SW (2011). aPKC phosphorylates NuMA-related LIN-5 to position the mitotic spindle during asymmetric division.. Nat Cell Biol.

[16] Hutterer A, Betschinger J, Petronczki M, Knoblich JA (2004). Sequential roles of Cdc42, Par-6, aPKC, and Lgl in the establishment of epithelial polarity during Drosophila embryogenesis.. Dev Cell.

[17] Morais-de-Sa E, Mirouse V, St. Johnston D (2010). aPKC phosphorylation of Bazooka defines the apical/lateral border in Drosophila epithelial cells.. Cell.

[18] Plant PJ, Fawcett JP, Lin DC, Holdorf AD, Binns K (2003). A polarity complex of mPar-6 and atypical PKC binds, phosphorylates and regulates mammalian Lgl.. Nat Cell Biol.

[19] Yamanaka T, Horikoshi Y, Sugiyama Y, Ishiyama C, Suzuki A (2003). Mammalian Lgl forms a protein complex with PAR-6 and aPKC independently of PAR-3 to regulate epithelial cell polarity.. Curr Biol.

[20] Lin D, Edwards AS, Fawcett JP, Mbamalu G, Scott JD (2000). A mammalian PAR-3-PAR-6 complex implicated in Cdc42/Rac1 and aPKC signalling and cell polarity.. Nat Cell Biol.

[21] Nagai-Tamai Y, Mizuno K, Hirose T, Suzuki A, Ohno S (2002). Regulated protein-protein interaction between aPKC and PAR-3 plays an essential role in the polarization of epithelial cells.. Genes Cells.

[22] Nishimura T, Kaibuchi K (2007). Numb controls integrin endocytosis for directional cell migration with aPKC and PAR-3.. Dev Cell.

[23] Smith CA, Lau KM, Rahmani Z, Dho SE, Brothers G (2007). aPKC-mediated phosphorylation regulates asymmetric membrane localization of the cell fate determinant Numb.. EMBO J.

[24] Atwood SX, Prehoda KE (2009). aPKC phosphorylates Miranda to polarize fate determinants during neuroblast asymmetric cell division.. Curr Biol.

[25] Djiane A, Yogev S, Mlodzik M (2005). The apical determinants aPKC and dPatj regulate Frizzled-dependent planar cell polarity in the Drosophila eye.. Cell.

[26] Hao Y, Du Q, Chen X, Zheng Z, Balsbaugh JL (2010). Par3 controls epithelial spindle orientation by aPKC-mediated phosphorylation of apical Pins.. Curr Biol.

[27] Banko MR, Allen JJ, Schaffer BE, Wilker EW, Tsou P (2011). Chemical Genetic Screen for AMPKα2 Substrates Uncovers a Network of Proteins Involved in Mitosis.. Mol Cell.

[28] Hindley AD, Park S, Wang L, Shah K, Wang Y (2004). Engineering the serine/threonine protein kinase Raf-1 to utilise an orthogonal analogue of ATP substituted at the N6 position.. FEBS Lett.

[29] Allen JJ, Lazerwith SE, Shokat KM (2005). Bio-orthogonal affinity purification of direct kinase substrates.. J Am Chem Soc.

[30] Allen JJ, Li M, Brinkworth CS, Paulson JL, Wang D (2007). A semisynthetic epitope for kinase substrates.. Nat Methods.

[31] Blethrow JD, Glavy JS, Morgan DO, Shokat KM (2008). Covalent capture of kinase-specific phosphopeptides reveals Cdk1-cyclin B substrates.. Proc Natl Acad Sci U S A.

[32] Westerfield M (1994). The Zebrafish Book.. Eugene: University of Oregon Press.

[33] Kimmel CB, Ballard WW, Kimmel SR, Ullmann B, Schilling TF (1995). Stages of embryonic development of the zebrafish.. Dev Dyn.

[34] Huang CJ, Tu CT, Hsiao CD, Hsieh FJ, Tsai HJ (2003). Germ-line transmission of a myocardium-specific GFP transgene reveals critical regulatory elements in the cardiac myosin light chain 2 promoter of zebrafish.. Dev Dyn.

[35] Link V, Shevchenko A, Heisenberg CP (2006). Proteomics of early zebrafish embryos.. BMC Dev Biol.

[36] Cravatt BF (2005). Kinase chemical genomics–a new rule for the exceptions.. Nat Methods.

